# *In Vitro* Activity of Sodium New Houttuyfonate Alone and in Combination with Oxacillin or Netilmicin against Methicillin-Resistant *Staphylococcus aureus*

**DOI:** 10.1371/journal.pone.0068053

**Published:** 2013-07-02

**Authors:** Xi Lu, Xinyi Yang, Xue Li, Yun Lu, Zhitao Ren, Longyin Zhao, Xinxin Hu, Jiandong Jiang, Xuefu You

**Affiliations:** 1 Institute of Medicinal Biotechnology, Chinese Academy of Medical Sciences and Peking Union Medical College, Beijing, China; 2 Beijing Standard Herbs Medical Science and Technology Development Co. LTD., Beijing, China; University Hospital Münster, Germany

## Abstract

**Background:**

*Staphylococcus aureus* can cause severe infections, including bacteremia and sepsis. The spread of methicillin-resistant *Staphylococcus aureus* (MRSA) highlights the need for novel treatment options. Sodium new houttuyfonate (SNH) is an analogue of houttuynin, the main antibacterial ingredient of *Houttuynia cordata* Thunb. The aim of this study was to evaluate *in vitro* activity of SNH and its potential for synergy with antibiotics against hospital-associated MRSA.

**Methodology:**

A total of 103 MRSA clinical isolates recovered in two hospitals in Beijing were evaluated for susceptibility to SNH, oxacillin, cephalothin, meropenem, vancomycin, levofloxacin, minocycline, netilmicin, and trimethoprim/sulfamethoxazole by broth microdilution. Ten isolates were evaluated for potential for synergy between SNH and the antibiotics above by checkerboard assay. Time-kill analysis was performed in three isolates to characterize the kill kinetics of SNH alone and in combination with the antibiotics that engendered synergy in checkerboard assays. Besides, two reference strains were included in all assays.

**Principal Findings:**

SNH inhibited all test strains with minimum inhibitory concentrations (MICs) ranging from 16 to 64 µg/mL in susceptibility tests, and displayed inhibition to bacterial growth in concentration-dependent manner in time-kill analysis. In synergy studies, the combinations of SNH-oxacillin, SNH-cephalothin, SNH-meropenem and SNH-netilmicin showed synergistic effects against 12 MRSA strains with median fractional inhibitory concentration (FIC) indices of 0.38, 0.38, 0.25 and 0.38 in checkerboard assays. In time-kill analysis, SNH at 1/2 MIC in combination with oxacillin at 1/128 to 1/64 MIC or netilmicin at 1/8 to 1/2 MIC decreased the viable colonies by ≥2log_10_ CFU/mL.

**Conclusions/Significance:**

SNH demonstrated *in vitro* antibacterial activity against 103 hospital-associated MRSA isolates. Combinations of sub-MIC levels of SNH and oxacillin or netilmicin significantly improved the *in vitro* antibacterial activity against MRSA compared with either drug alone. The SNH-based combinations showed promise in combating MRSA.

## Introduction

Over the past few decades, antimicrobial resistance has been recognized as a major public health problem. Since it was first recovered from patients in southern England in 1961 [1], methicillin-resistant *Staphylococcus aureus* (MRSA) has been reported worldwide and has become one of the leading causes of hospital-associated and community-acquired infections [2]. In China, infections caused by MRSA in healthcare institutions have increased greatly in the past 20 years. It was reported that mean prevalence of MRSA in 16 medical centers in 12 cities across China had reached 50.4% by 2005 [3]. Treatment options for MRSA are limited and less effective than options available for methicillin-susceptible *Staphylococcus aureus* (MSSA) infections [4]. This emphasizes the importance of developing more therapeutic options including novel antimicrobials and combinations of existing drugs to combat MRSA.

**Figure 1:**
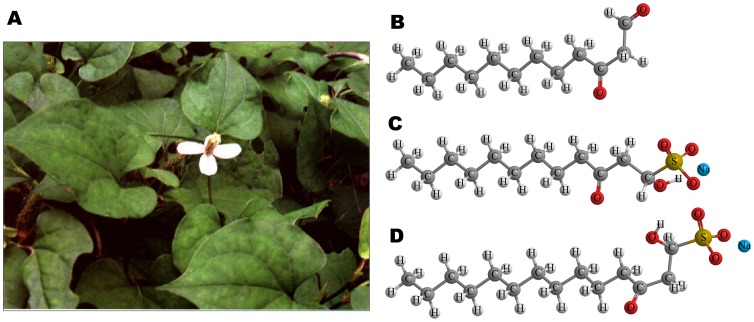
*Houttuynia cordata* Thunb and related compounds. A) *Houttuynia cordata* Thunb (picture of the plant is from Chinese Pharmacopoeia Commission, 9^th^ edition [8]), B) Houttuynin, C) Sodium houttuyfonate and D) Sodium new houttuyfonate.

Historically, plants have provided us with a good source of antimicrobial agents. Drugs of plant origin including artesunate, berberine, and quinine are still effective in some cases in treating infectious diseases [5], [6], [7]. *Houttuynia cordata* Thunb (Figure 1. A) is a common vegetable consumed in Southwest China as well as an herb used in traditional Chinese medicine for hundreds of years [8]. In April 2003, it was approved by the State Administration of Traditional Chinese Medicine of the People's Republic of China as one of the component herbs in a Chinese herb formula to prevent severe acute respiratory syndrome (SARS) [9]. Recently, several studies also provide scientific data to support and unveil its antibacterial [10], [11], anti-inflammatory [12], and antiviral [13] activities. Houttuynin (decanoyl acetaldehyde, C_12_H_22_O_2_, MW = 198.3) is the main antibacterial ingredient in volatile oil of *Houttuynia cordata* Thunb. As houttuynin is chemically unstable, sodium houttuyfonate (SH, sodium 1-hydroxy-3-oxododecane-1-sulfonate, C_12_H_23_NaO_5_S, MW = 302.4) and sodium new houttuyfonate (SNH, sodium lauroyl-α-hydroxyethyl sulfonate, C_14_H_27_NaO_5_S, MW = 330.4), analogue of sodium houttuyfonate, were synthesized and have been approved by China Food and Drug Administration to be used in clinical practice mainly in the formulations of tablets and injection [14], [15]. Due to its improved chemical and pharmacological properties, SNH has replaced houttuynin, and to a large extent SH, as an effective therapeutic agent for respiratory infections and inflammatory diseases such as acute or chronic bronchitis and pneumonia in clinical settings [16], [17]. The structures of the three compounds are shown in Figure 1. Earlier studies indicated that a variety of bacteria could be inhibited by SNH with gram-positive bacteria showing a higher sensitivity to the compound than gram-negative bacteria *in vitro* [18], [19]. However, no report has specially focused on activity of SNH against MRSA. In this study, we examined the *in vitro* activity of SNH and its potential for synergy when combined with antibiotics against a collection of hospital-associated MRSA (HA-MRSA) isolates recovered from various clinical samples in recent years.

## Materials and Methods

### Ethics Statement

No permits were required for the described study, which complied with all relevant regulations.

### Bacterial Strains

A total of 103 MRSA strains collected from a variety of clinical samples in two hospitals in Beijing between 2005 and 2010 were used in this study. All MRSA isolates were identified by automated antimicrobial susceptibility test systems (BD or bioMerieux systems) in hospital laboratories and were further confirmed by VITEK 2-COMPACT system (bioMerieux, Marcy I'Etoile, France) and the standard oxacillin agar dilution method recommended by Clinical and Laboratory Standards Institute (CLSI, formerly NCCLS) in our laboratory. In addition, genotypic features of these isolates were characterized by multiplex PCR for the *mecA* gene identification and SCC*mec* typing. Relevant characteristics of the MRSA isolates used in this study are presented in Table 1. Generally, SCC*mec* type III is the predominant type in HA-MRSA strains in China [20]. Two ATCC (American Tissue Culture and Collection) MRSA strains, ATCC33591 and Mu 50 (also referred to as ATCC 700699, vancomycin-intermediate *S. aureus*) were used as quality control strains.

**Table 1:** Characteristics of 103 hospital-associated MRSA isolates used in this study.

origin	oxacillin- resistantphenotype No. (%)	No. of isolates positivefor *mecA* (% of total)	SCC*mec* type (No. of isolates)				
			I	II	III	IV	V
Upper respiratory tract infection	26 (100%)	26 (100%)	0	3	23	0	0
Pneumonia	21 (100%)	21 (100%)	0	2	18	1	0
Skin and soft tissue wounds	17 (100%)	17 (100%)	0	4	13	0	0
Urinary tract infection	18 (100%)	18 (100%)	0	5	13	0	0
Bacteremia	15 (100%)	15 (100%)	0	0	15	0	0
Pyothoroax	4 (100%)	4 (100%)	0	0	4	0	0
Meningitis	2 (100%)	2 (100%)	0	0	2	0	0
Total (%)	103 (100%)	103 (100%)	0 (0%)	14 (13.6%)	88 (85.4%)	1 (1.0%)	0 (0%)

### Antimicrobial Agents and Medium

Sodium New Houttuyfonate was provided by Beijing Standardherbs Medical Science & Technology Development Co. LTD. Eight antimicrobial agents, including oxacillin (OXA), cephalothin (CEF), meropenem (MEM), vancomycin (VAN), levofloxacin (LVX), minocycline (MIN), netilmicin (NET), and trimethoprim-sulfamethoxazole (SXT) were commercially purchased. SNH was dissolved in distilled water. Stock solutions of antibiotics were prepared in solvents and diluents recommended by CLSI [21] based on their actual purity or potency and sterilized through 0.22 µm filters before use. Test solutions with different concentrations of SNH and the antimicrobials were obtained by two-fold serial dilutions with cation-adjusted Mueller-Hinton (CAMH) broth (BD, Cockeysville, MD).

CAMH broth was used for all susceptibility testing, checkerboard testing and time-kill analysis. Colony counts were determined using tryptic soy agar (TSA; BD, Cockeysville, MD) plates.

### Susceptibility Testing

The minimum inhibitory concentrations (MICs) of the antibacterial agents for all 103 clinical isolates were determined by broth microdilution method according to CLSI guidelines [22]. Wells of 96-well microtiter plates (Nunc, Thermo Fisher Scientific Inc., Roskilde) were inoculated with 100 µL of CAMH broth containing serial-diluted antimicrobials and a final inoculum of 5×10^5^ CFU/mL. The concentrations of SNH ranged from 1 µg/mL to 128 µg/mL. The concentration ranges for other antibiotics were as follows: 0.03 µg/mL to 128 µg/mL for MEM, VAN, LVX, MIN, and NET; 0.06 µg/mL to 1024 µg/mL for OXA and CEF; 0.008/0.15 µg/mL to 32/608 µg/mL for SXT. Testing of oxacillin was performed in CAMH broth supplemented with 2% NaCl. The microtiter plates were incubated at 35°C for 24 h. The MIC was defined as the lowest concentration of an antimicrobial agent that prevented turbidity. All MIC determinations were performed in duplicate.

### Time-kill Curves of SNH

Kill kinetics of SNH was determined by time-kill experiments for five MRSA strains (three clinical isolates MRSA 5–20, MRSA 6–29 and MRSA 8–36 and two reference strains ATCC 33591 and Mu50) according to the method described by Verma et al. with slight modifications [23]. An overnight culture was diluted with CAMH broth in a total volume of 30 mL containing an inoculum of 2×10^6^ CFU/mL in a 250 mL flask for each strain. Distilled water or SNH was added to yield concentrations of 0×, 1/4×, 1/2×, 1×, 2×, and 4×MIC in the broth at standard inocula.

Viability counts were performed at 0, 2, 4, 6, 8 and 24 h of incubation at 37°C by plating 0.1 mL undiluted and 10-fold serial diluted samples onto TSA plates in duplicate. Drug carryover effect was eliminated by saline and agar dilution. The experiments were performed three times on different days and the results were presented as mean and standard deviation. The time–kill curves were recorded as log_10_ reductions in bacterial counts within a specific time period. Bactericidal activity was defined as ≥3 log_10_CFU/mL reduction (99.9% kill) in colony count from the starting inoculum. The detection limit was 2log_10_CFU/mL.

### Studies for Synergy

#### Checkerboard assay

Eight combinations including SNH-OXA, SNH-CEF, SNH-MEM, SNH-VAN, SNH-LVX, SNH-MIN, SNH-NET, and SNH-SXT were evaluated on 10 randomly selected MRSA isolates and two quality control strains (ATCC 33591 and Mu 50) using microdilution checkerboard technique [23], [24]. In brief, a final inoculum of 5×10^5^ CFU/mL was added to wells of 96-well microtiter plates containing two-fold diluted SNH and the other antimicrobial in CAMH broth. After incubation at 35°C for 24 h, the combined effect of SNH with each antimicrobial was analyzed by calculation of the fractional inhibitory concentration index (FICI) using the following equation: FICI = (MIC of drug A in the combination/MIC of drug A alone)+(MIC of drug B in the combination/MIC of drug B alone). The antimicrobial combination was defined to be synergistic when the FICI was ≤0.5; indifferent when 0.5< FICI <4; antagonistic when FICI ≥4 [23], [24]. The experiments were performed in duplicate on different days.

#### Time-kill curve analysis of SNH-OXA, SNH-NET and SNH-MEM combinations

Time-kill assays were performed for ATCC 33591, Mu 50, and the clinical isolates MRSA 5–20, MRSA 6–29 and MRSA 8–36 to evaluate the killing dynamics of SNH-OXA, SNH-NET and SNH-MEM, the combinations of interest. The time-kill for the combination study was similar to that described in the time kill assays of individual use of SNH. Antimicrobial-free inoculation of each strain was included as growth control. SNH was combined with oxacillin, meropenem or netilmicin and tested at concentrations below MIC of each drug. The concentrations of antimicrobials used for different strains were 1/2 MIC or MIC for SNH, 1/128 to 1/64 MIC for OXA, 1/8 to 1/2 MIC for NET and 1/16 to 1/4 MIC for MEM. Colony counts were determined at 0, 2, 4, 6, 8, 24, 48, and 72 h. Synergy was defined as ≥2 log_10_ decrease in CFU/mL between the combination and its most active constituent at 72 h, with the number of surviving organisms in the combination at ≥2log_10_ CFU/mL below the starting inoculum.

## Results

### Susceptibility Testing

The susceptibility results of all antimicrobials tested against 103 MRSA isolates and 2 reference strains are presented in Table 2. SNH inhibited all the test MRSA strains regardless of their origins and SCC*mec* types at concentrations ranging from 16 to 64 µg/mL. Both MIC_50_ and MIC_90_ values of SNH were 32 µg/mL. The MICs of the antibiotics for the quality control strains ATCC 33591 and Mu 50 were within the expected ranges. Most of the strains were resistant to three β-lactams (OXA, CEF and MEM), LVX, MIN, NET, or all of the six antibiotics. Among the eight antibiotics, only vancomycin and trimethoprim-sulfamethoxazole showed favorable antibacterial activity, except for the vancomycin-intermediate strain Mu 50.

**Table 2:** Susceptibility of MRSA strains to SNH and eight antibiotics.

Antimicrobials	MIC of control strains (µg/mL)		MIC of 103 clinical isolates (µg/mL)			Susceptible/total a (% Susceptibility)
	ATCC 33591	Mu50	Range	50%	90%	
SNH	32	64	16–64	32	32	NA^b^
OXA	256	512	8–512	512	512	0/103(0)
CEF	32	128	1–256	64	256	3/103(2.9)
MEM	16	32	2–64	32	64	4/103(3.9)
VAN	1	8	0.5–2	1	1	103/103(100)
LVX	0.25	16	0.25–64	16	32	5/103 (4.9)
MIN	8	8	0.25–32	8	16	60/103(58.3)
NET	4	16	0.5–128	16	64	8/103(7.8)
SXT	1/19	0.25/4.8	0.03/0.57–4/76	0.125/2.38	1/19	97/103(94.2)

a The susceptibility was determined according to standards recommended by CLSI: ≤2 µg/mL for oxacillin, ≤8 µg/mL for cephalothin, ≤4 µg/mL for meropenem, ≤2 µg/mL for vancomycin, ≤1 µg/mL for levofloxacin, ≤4 µg/mL for minocycline, ≤8 µg/mL for netilmicin, and ≤2 µg/mL/38 µg/mL for trimethoprim-sulfamethoxazole.

b NA, not applicable.

### Time-kill Curve Analysis of SNH

**Figure 2:**
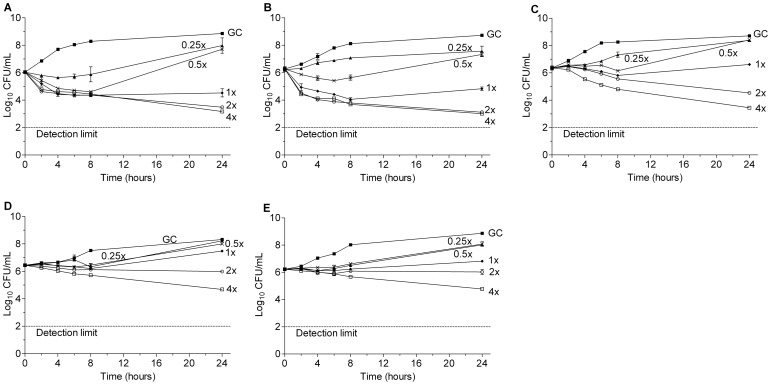
Activity of SNH at different concentrations in a time-kill analysis against MRSA strains. A) MRSA 5–20 (MIC = 32 µg/mL); B) MRSA 6–29 (MIC = 32 µg/mL); C) MRSA 8–36 (MIC = 32 µg/mL); D) ATCC33591 (MIC = 32 µg/mL); E) Mu50 (MIC = 64 µg/mL); ▪, GC, growth control; ▴, 1/4×MIC; ×, 1/2×MIC; ⧫, 1×MIC; ○, 2×MIC; □, 4×MIC.

The time–kill curves of SNH for three MRSA clinical isolates and two reference strains are displayed in Figure 2. Concentration-dependent killing and significant reductions in the viable bacterial counts were observed after 24 h exposure of the bacteria to SNH at concentrations above MIC. The maximum reductions in viable counts were 1.5 to 3 log_10_ CFU/mL for 4×MIC of SNH when compared with the initial inocula; the antibacterial effect of SNH on the five strains was determined to be bacteriostatic or marginal bactericidal at test concentrations.

### Studies for Synergy

#### Checkerboard assay

The results of the checkerboard analysis are summarized in Table 3 (detailed MIC and FICI values are presented in Table S1). Synergistic interactions were noted between SNH and β-lactams as well as SNH and NET based on FIC indices for these combinations. SNH-OXA, SNH-CEF, SNH-MEM and SNH-NET demonstrated synergism in 11, 9, 12 and 12 of the 12 MRSA strains, respectively. The inhibitory concentrations of OXA, CEF, MEM, and NET against most strains decreased significantly to 1/32–1/4 MIC when combined with 1/8 to1/4 MIC of SNH (2–16 µg/mL). According to the FIC indices, combinations of SNH-VAN and SNH-LVX displayed indifferent effect in all the 12 strains; SNH-MIN and SNH-SXT exhibited indifferent effect in 10 strains and synergistic effect in 2 strains of the 12 strains tested. No evidence of antagonism was noted for the eight SNH-based combinations against any of the strains tested.

**Table 3:** Effects of SNH in combination with antibiotics against 12 MRSA strains in checkerboard assay.

Antimicrobial combination	Range of FICI	Median of FICI	Checkerboard effect (No. of isolates)		
			Synergy	indifference	Antagonism
SNH-OXA	0.28–0.56	0.38	11	1	0
SNH-CEF	0.31–0.63	0.38	9	3	0
SNH-MEM	0.16–0.38	0.25	12	0	0
SNH-VAN	0.63–2	1	0	12	0
SNH-LVX	0.53–2	1	0	12	0
SNH-MIN	0.5–0.75	0.63	2	10	0
SNH-NET	0.25–0.5	0.38	12	0	0
SNH-SXT	0.5–2	0.69	2	10	0

#### Time-kill curve analysis of SNH-OXA, SNH-NET and SNH-MEM combinations

**Figure 3:**
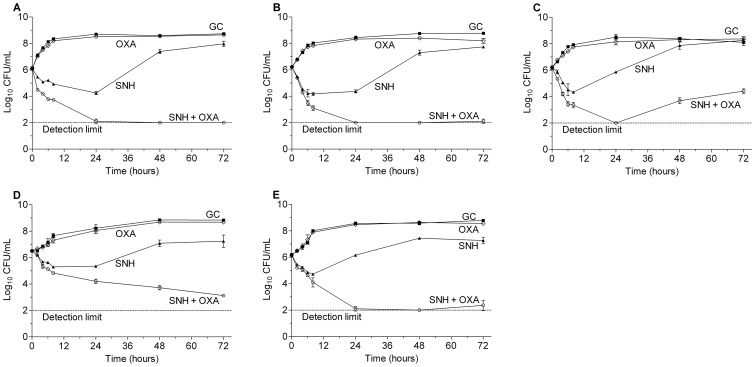
Sub-MIC levels of SNH and oxacillin alone and in combination against MRSA strains. A) MRSA 5–20, 1/2×MIC SNH-1/64×MIC OXA (MIC of SNH = 32 µg/mL, MIC of OXA = 128 µg/mL); B) MRSA 6–29, 1/2×MIC SNH-1/64×MIC OXA (MIC of SNH = 32 µg/mL, MIC of OXA = 256 µg/mL); C) MRSA 8–36, 1/2×MIC SNH-1/128×MIC OXA (MIC of SNH = 32 µg/mL, MIC of OXA = 512 µg/mL); D) ATCC 33591 1/2×MIC SNH-1/64×MIC OXA (MIC of SNH = 32 µg/mL, MIC of OXA = 256 µg/mL); E) Mu 50, 1/2×MIC SNH-1/128×MIC OXA (MIC of SNH = 64 µg/mL, MIC of OXA = 512 µg/mL); ▪, GC, growth control; ▴, SNH; ◊, OXA; ○, combination of SNH and OXA.

**Figure 4:**
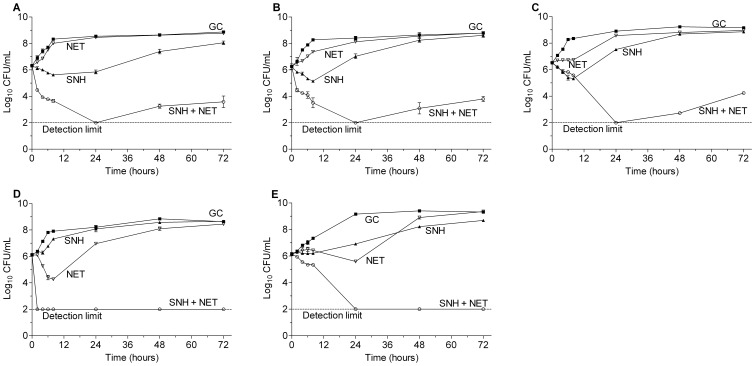
Sub-MIC levels of SNH and netilmicin alone and in combination against MRSA strains. A) MRSA 5–20, 1/2×MIC SNH-1/4×MIC NET (MIC of SNH = 32 µg/mL, MIC of NET = 16 µg/mL); B) MRSA 6–29, 1/2×MIC SNH-1/2×MIC NET (MIC of SNH = 32 µg/mL, MIC of NET = 8 µg/mL); C) MRSA 8–36, 1/2×MIC SNH-1/8×MIC NET (MIC of SNH = 32 µg/mL, MIC of NET = 64 µg/mL); D) ATCC 33591 1/2×MIC SNH-1/4×MIC NET (MIC of SNH = 32 µg/mL, MIC of NET = 4 µg/mL); E) Mu 50, 1/2×MIC SNH-1/4×MIC NET (MIC of SNH = 64 µg/mL, MIC of NET = 16 µg/mL); ▪, GC, growth control; ▴, SNH; ▽, NET; ○, combination of SNH and NET.

The time-kill results of SNH-OXA, SNH-NET and SNH-MEM against the five MRSA strains are displayed in Figure 3, 4 and S1, respectively. At the concentrations of sub-MIC levels tested, the three antibiotics used alone showed little activity on bacterial growth. SNH alone showed weak inhibitory effect on the bacteria but was followed by bacterial regrowth after 8 to24 h of incubation. In contrast, the combinations of SNH with OXA, MEM or NET resulted in potent synergistic effect on all the test strains. The combinations greatly reduced the viable counts of bacteria by ≥2log_10_ CFU/mL when compared with any of the most active agent used individually. The synergistic effect could still be observed at 72 h for some of the strains in certain combinations. In addition, the three combinations reduced the viable count by >3log_10_ when compared with the starting inoculum and bactericidal effect was demonstrated.

## Discussion

SNH contains a hydrophilic sulfinyl head and a hydrophobic alkyl tail with 12 carbon atoms, which is considered to be surfactant-like structure. Although antibacterial mechanism of SNH remains unknown, it was indicated that SNH exerted its antimicrobial effect mainly through binding of non-polar tail group to bacterial hydrophobic membrane proteins or cytoplasmic enzymes [18], [25], [26]. The hydrophobic lipid bilayer of cell membrane might be another target of aliphatic chain of SNH, but it seems that a repulsive force between the negatively charged membrane and the anionic hydrophilic moiety of SNH may weaken the potential interaction [27]. This might be an explanation of higher susceptibility of gram positive bacteria to SNH than gram negative bacteria, which have higher negative charge on the outer membrane due to presence of abundant anion charged phospholipids and lipopolysaccharides [28]. In addition, a latest report showed that treatment of SH, analogue of SNH, decreased transcription of some autolysins and inhibited Triton X-100-induced autolysis in *S. aureus in vitro*. Although correlation between inhibition of autolysis of *S. aureus* and antimicrobial effect of SH needs further elucidation, the findings provided a possible explanation of response of *S. aureus* to SNH-like compounds [29]. Overall, the enhanced antibacterial effects of SNH-based combinations may be attributed to molecular property of SNH which disrupted integrity and function of cell membrane. Since membrane-mediated processes involved in cell wall biosynthesis, cell permeability and drug efflux are important sources of resistance of MRSA to some antimicrobials, it seems reasonable to hypothesize that MRSA will be sensitized to antimicrobials by disrupting the normal barrier function of the cell membrane. Further work is required to identify the exact targets of SNH and understand the mechanisms involved in the interactions between SNH and β-lactams or aminoglycosides.

SNH was reported to exhibit potent inhibitory activity against *Staphylococcus aureus*, *Staphylococcus epidermidis*, and *Klebsiella pneumoniae*, etc. [30]. This study evaluated activity of SNH against MRSA and its potential for synergistic effect when combined with antibiotics. SNH has both MIC_50_ and MIC_90_ values of 32 µg/mL for 103 MRSA strains and displayed growth inhibitory or marginal killing activity against MRSA in a concentration-dependent manner. In another set of experiments, we demonstrated that there was no significant difference between MSSA and MRSA on susceptibility to SNH (Table S1) and characteristics of dynamic time-kill curves of SNH (Figure S2). As a result, it is unlikely that there is cross-resistance between SNH and β-lactams.

SNH in combination with OXA exhibited synergistic effect for all the five MRSA strains tested in time-kill analysis. Similarly, it was also demonstrated that the SNH-OXA combination showed synergism on four MSSA strains tested in another set of time-kill assays conducted to observe its action on MSSA (Figure S3). Besides SNH-OXA combination, SNH-NET and SNH-MEM combinations also showed synergistic effect on MRSA strains. Stressed by SNH-based combinations, the viability of the organisms was largely depressed. Although regrowth of bacteria was seen for some strains in certain combinations, it was mild and only occurred after 24 h. These findings reflect the gradual and stable antibacterial effect of these drug combinations. As use of MEM is reserved to treatment of infections caused by multidrug-resistant (MDR) gram negative bacteria in clinical settings, application of SNH-MEM combination on gram positive cocci including MRSA doesn’t seem feasible. It might be interesting to test this combination on MDR gram negative bacteria.

Considering that oxacillin can combine with other antimicrobial agents to restore its utility against staphylococci [31], while netilmicin was also reported to be effective on MRSA infection when used in a drug combination [32], [33], the findings reported here suggest that in vitro combinations of subinhibitory concentrations of SNH-OXA and SNH-NET could be beneficial inhibiting MRSA. However, further investigations, including pharmacokinetic/pharmacodynamic studies need to be conducted to determine if the in vivo activity of SNH-based combinations exists. If so, patients could potentially benefit from these findings.

## Supporting Information

Figure S1 **Sub-MIC levels of SNH and meropenem alone and in combination against MRSA strains.** A. MRSA 5–20, 1/2×MIC SNH-1/4×MIC MEM (MIC of SNH = 32 µg/mL, MIC of MEM = 16 µg/mL); B. MRSA 6–29, 1/2×MIC SNH-1/8×MIC MEM (MIC of SNH = 32 µg/mL, MIC of MEM = 32 µg/mL); C. MRSA 8–36, 1/2×MIC SNH-1/16×MIC MEM (MIC of SNH = 32 µg/mL, MIC of MEM = 64 µg/mL); D. ATCC 33591 1/2×MIC SNH-1/4×MIC MEM (MIC of SNH = 32 µg/mL, MIC of MEM = 16 µg/mL); E. Mu 50, 1×MIC SNH-1/4×MIC MEM (MIC of SNH = 64 µg/mL, MIC of MEM = 32 µg/mL); ▪, GC, growth control; ▴, SNH; □, MEM; ○, combination of SNH and MEM.
(TIF)

Figure S2 **Activity of SNH at different concentrations in time-kill analysis against MSSA strains.** A. MSSA 7–3 (MIC = 32 µg/mL); B. MSSA 7–4 (MIC = 32 µg/mL); C. MSSA 8–8 (MIC = 32 µg/mL); D. ATCC29213 (MIC = 32 µg/mL); ▪, GC, growth control; ▴, 1/4×MIC; ×, 1/2×MIC; ⧫, 1×MIC; ○, 2×MIC; □, 4×MIC.
(TIF)

Figure S3 **Sub-MIC levels of SNH and oxacillin alone and in combination against MSSA strains.** A. MSSA 7-3, 1/2×MIC SNH-1/4×MIC OXA(MIC of SNH = 32 µg/mL, MIC of OXA = 0.5 µg/mL); B. MSSA 7-4, 1/2×MIC SNH-1/4×MIC OXA (MIC of SNH = 32 µg/mL, MIC of OXA = 0.5 µg/mL); C. MSSA 8-8, 1/2×MIC SNH-1/4×MIC OXA (MIC of SNH = 32 µg/mL, MIC of OXA = 0.5 µg/mL); D. ATCC29213 1/2×MIC SNH-1/4×MIC OXA (MIC of SNH = 32 µg/mL, MIC of OXA = 0.5 µg/mL); ▪, GC, growth control; ▴, SNH; ◊, OXA; ○, combination of SNH and OXA.
(TIF)

Table S1 **MICs and FICI of SNH and eight antibiotics against 12 MRSA strains in checkerboard assay.**
(PDF)

Table S2 **Susceptibility of 20 MSSA strains to SNH and oxacillin.**
(PDF)

## References

[1] Patricia MJ (1961) “Celbenin”-resistant Staphylococci. British Medical Journal 14: 124–125.

[2] Moellering RC Jr (2012) MRSA: the first half century. J Antimicrob Chemother 67: 4–11. 22010206 10.1093/jac/dkr437

[3] Wang H, Liu Y, Sun H, Xu Y, Xie X, et al (2008) In vitro activity of ceftobiprole, linezolid, tigecycline, and 23 other antimicrobial agents against Staphylococcus aureus isolates in China. Diagn Microbiol Infect Dis 62: 226–229. 18653301 10.1016/j.diagmicrobio.2008.06.003

[4] Gould IM, David MZ, Esposito S, Garau J, Lina G, et al (2012) New insights into meticillin-resistant Staphylococcus aureus (MRSA) pathogenesis, treatment and resistance. Int J Antimicrob Agents 39: 96–104. 22196394 10.1016/j.ijantimicag.2011.09.028

[5] Adjuik M, Agnamey P, Babiker A, Baptista J, Borrmann S, et al (2004) Artesunate combinations for treatment of malaria: meta-analysis. Lancet 363: 9–17. 14723987 10.1016/s0140-6736(03)15162-8

[6] Faiz MA, Bin Yunus E, Rahman MR, Islam F, Hoque MG, et al (2005) Artesunate versus quinine for treatment of severe falciparum malaria: a randomised trial. Lancet 366: 717–725. 16125588 10.1016/S0140-6736(05)67176-0

[7] Rabbani GH, Butler T, Knight J, Sanyal SC, Alam K (1987) Randomized controlled trial of berberine sulfate therapy for diarrhea due to enterotoxigenic Escherichia coli and Vibrio cholerae. J Infect Dis 155: 979–984. 3549923 10.1093/infdis/155.5.979

[8] Chinese Pharmacopoeia Commission (2010) Chinese Material Medica and Prepared Slices of Chinese Crude Drugs. Pharmacopoeia of the People’s Republic of China.9 ed. Beijing, China: China Medical Science Press. 208–209.

[9] State Administration of Traditional Chinese Medicine of the People's Republic of China website. Available: http://www.satcm.gov.cn/zhuanti/jbfz/20060901/100052.shtml. Accessed 2013 Jun 5.

[10] Lu H, Wu X, Liang Y, Zhang J (2006) Variation in chemical composition and antibacterial activities of essential oils from two species of Houttuynia Thunb. Chem Pharm Bull 54: 936–940. 16819207 10.1248/cpb.54.936

[11] Kim GS, Kim DH, Lim JJ, Lee JJ, Han DY, et al (2008) Biological and antibacterial activities of the natural herb Houttuynia cordata water extract against the intracellular bacterial pathogen salmonella within the RAW 264.7 macrophage. Biol Pharm Bull 31: 2012–2017. 18981565 10.1248/bpb.31.2012

[12] Lu HM, Liang YZ, Yi LZ, Wu XJ (2006) Anti-inflammatory effect of Houttuynia cordata injection. J Ethnopharmacol 104: 245–249. 16213118 10.1016/j.jep.2005.09.012 PMC7127264

[13] Hayashi K, Kamiya M, Hayashi T (1995) Virucidal effects of the steam distillate from Houttuynia cordata and its components on HSV-1, influenza virus, and HIV. Planta Med 61: 237–241. 7617766 10.1055/s-2006-958063

[14] Chinese Pharmacopoeia Commission (1995) Pharmacopoeia of the People’s Republic of China. Beijing, China: Chemical Industry Press. 411–412.

[15] China Food and Drug Administration website. Available: http://www.sda.gov.cn/bzzzmd9/50.htm. Accessed 2013 Jun 5.

[16] Liang L (2006) Observation of sodium new houttuyfonat injection in the treatment of children acute upper respiratory infection. Shenzhen Journal of Integrated Traditional Chinese and Western Medicine 16: 36–38.

[17] Yu X, Chen B, Zhu W (2005) Clinical observation on the treatment of bronchiolitis with nebulized-inhalation of sodium new houttuyfonate injection in 50 cases. Tianjin Pharmacy 17: 39–40.

[18] Ye XL, Li XG, Yuan LJ, He HM (2006) Relationship between the antibacterial and immunological activities of houttuyfonate homologues and their surface activities. J Asian Nat Prod Res 8: 327–334. 16864443 10.1080/10286020500035243

[19] Ye XL, Xu L, Li XG, Chen Z, Zhang BS, et al (2009) Antibacterial mechanism of houttuyfonate homologues against Bacillus subtilis. Colloids and Surfaces a-Physicochemical and Engineering Aspects 350: 130–135.

[20] Liu Y, Wang H, Du N, Shen E, Chen H, et al (2009) Molecular evidence for spread of two major methicillin-resistant Staphylococcus aureus clones with a unique geographic distribution in Chinese hospitals. Antimicrob Agents Chemother 53: 512–518. 19029328 10.1128/AAC.00804-08 PMC2630620

[21] Clinical and Laboratory Standards Institute (2007) Performance standards for antimicrobial susceptibility testing; seventeenth informational supplement. CLSI document M100-S17. Wayne, Pennsylvania, USA.

[22] Clinical and Laboratory Standards Institute (2012) Methods for Dilution Antimicrobial Susceptibility Tests for Bacteria That Grow Aerobically; Approved Standard. CLSI document M07-A9.9 ed. Wayne, Pennsylvania, USA.

[23] Verma P (2007) Methods for determining bactericidal activity and antimicrobial interactions synergy testing, time-kill curves, and population analysis. In: Schwalbe R, Steele-Moore L, Goodwin AC, editors. Antimicrobial susceptibility testing protocols. Roca Raton, Florida, USA: CRC Press. 275–298.

[24] Pillai S, Moellering R, Jr Eliopoulos G (2005) Antimicrobial Combinations. In: Lorian V, editor. Antibiotics in Laboratory Medicine. 5 ed. Philadelphia, Pennsylvania, USA: Lippincott Williams and Wilkins. 365–440.

[25] Ye X, Li X, Yuan L, Ge L, Zhang B, et al (2007) Interaction of houttuyfonate homologues with the cell membrane of gram-positive and gram-negative bacteria. Colloids and Surfaces A: Physicochemical and Engineering Aspects 301: 412–418.

[26] Ye X, Xu L, Li X, Chen Z, Zhang B, et al (2009) Antibacterial mechanism of houttuyfonate homologues against Bacillus subtilis. Colloids and Surfaces A: Physicochemical and Engineering Aspects 350: 130–135.

[27] Li Z, He X, Zuo H (2009) Synthesis of novel analogues on the α-carbon of houttuyfonate and SAR analysis of antibacterial activity with MOPAC. Medicinal Chemistry Research 18: 362–372.

[28] Savage PB, Li C, Taotafa U, Ding B, Guan Q (2002) Antibacterial properties of cationic steroid antibiotics. FEMS Microbiol Lett 217: 1–7. 12445638 10.1111/j.1574-6968.2002.tb11448.x

[29] Liu G, Xiang H, Tang X, Zhang K, Wu X, et al (2011) Transcriptional and functional analysis shows sodium houttuyfonate-mediated inhibition of autolysis in Staphylococcus aureus. Molecules 16: 8848–8865. 22019573 10.3390/molecules16108848 PMC6264171

[30] Qiu C, Xiao X, Lei H, Zhang Z, Feng X, et al (1979) Investigation of efficacy of sythesized sodium neo-houttuyfonate in clinical settings. New Medicine 10: 601–602.

[31] Jenkins RE, Cooper R (2012) Synergy between oxacillin and manuka honey sensitizes methicillin-resistant Staphylococcus aureus to oxacillin. J Antimicrob Chemother 67: 1405–1407. 22382468 10.1093/jac/dks071

[32] Nasu M, Goto Y, Yamasaki T, Hara K, Kohno S, et al (1994) Clinical studies on the time-difference combination therapy with netilmicin and minocycline in methicillin-resistant Staphylococcus aureus infections. Jpn J Antibiot 47: 1305–1317. 7807692

[33] Maesaki S, Mashimoto H, Araki J, Asai S, Sawatari K (1994) A case of MRSA sepsis treated by the sequential combination therapy netilmycin and minocycline. Kansenshogaku Zasshi 68: 1523–1526. 7876675 10.11150/kansenshogakuzasshi1970.68.1523

