# Supplementary figures and images for: In Vitro Activity of Sodium New Houttuyfonate Alone and in Combination with Oxacillin or Netilmicin against Methicillin-Resistant Staphylococcus aureus
Source: PLoS One. 2013 Jul 2;8(7):e68053. doi: 10.1371/journal.pone.0068053 (PMC3699466; doi:10.1371/journal.pone.0068053)

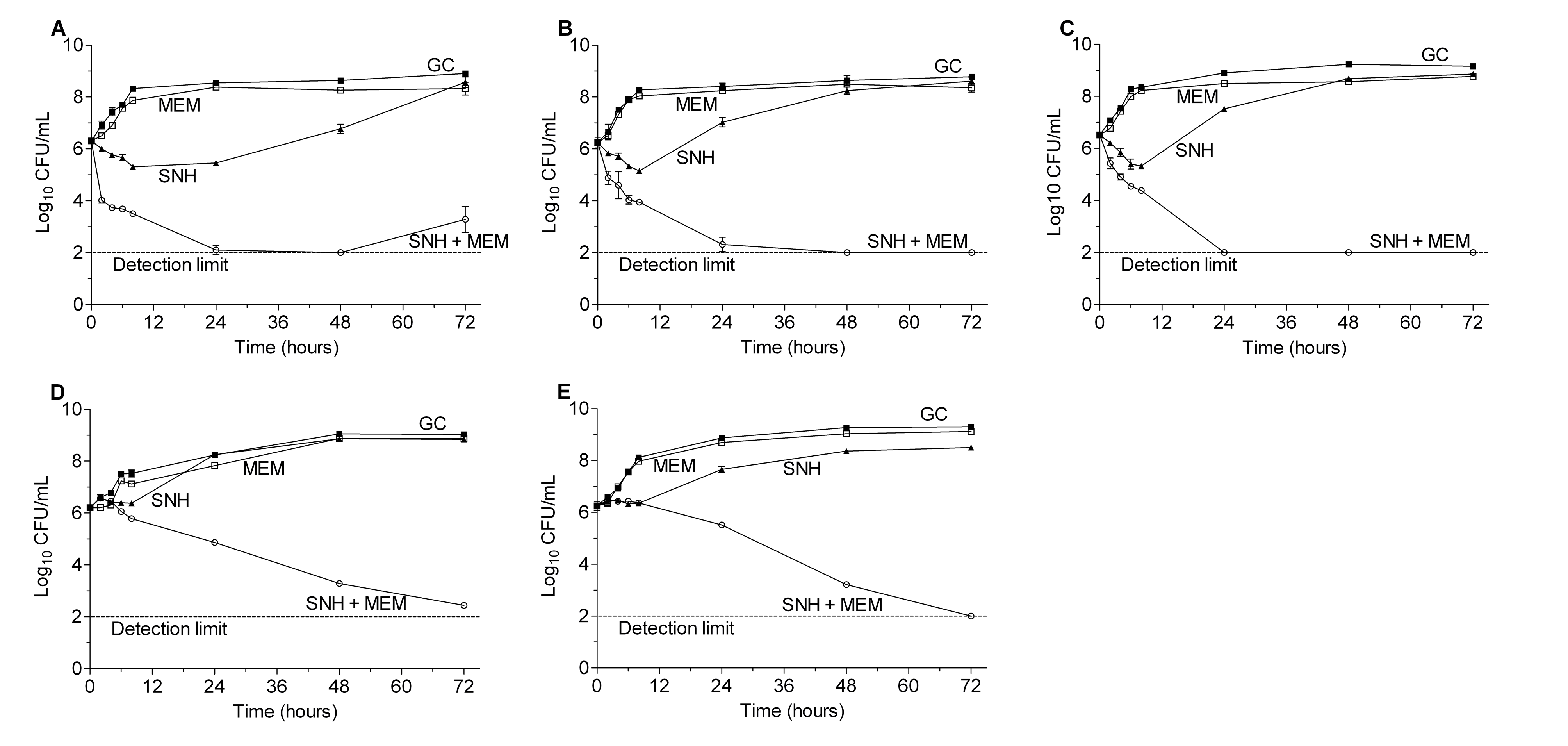

Supplement: Figure S1 — Sub-MIC levels of SNH and meropenem alone and in combination against MRSA strains. A. MRSA 5–20, 1/2×MIC SNH-1/4×MIC MEM (MIC of SNH = 32 µg/mL, MIC of MEM = 16 µg/mL); B. MRSA 6–29, 1/2×MIC SNH-1/8×MIC MEM (MIC of SNH = 32 µg/mL, MIC of MEM = 32 µg/mL); C. MRSA 8–36, 1/2×MIC SNH-1/16×MIC MEM (MIC of SNH = 32 µg/mL, MIC of MEM = 64 µg/mL); D. ATCC 33591 1/2×MIC SNH-1/4×MIC MEM (MIC of SNH = 32 µg/mL, MIC of MEM = 16 µg/mL); E. Mu 50, 1×MIC SNH-1/4×MIC MEM (MIC of SNH = 64 µg/mL, MIC of MEM = 32 µg/mL); ▪, GC, growth control; ▴, SNH; □, MEM; ○, combination of SNH and MEM. (TIF) [file pone.0068053.s001.tif]

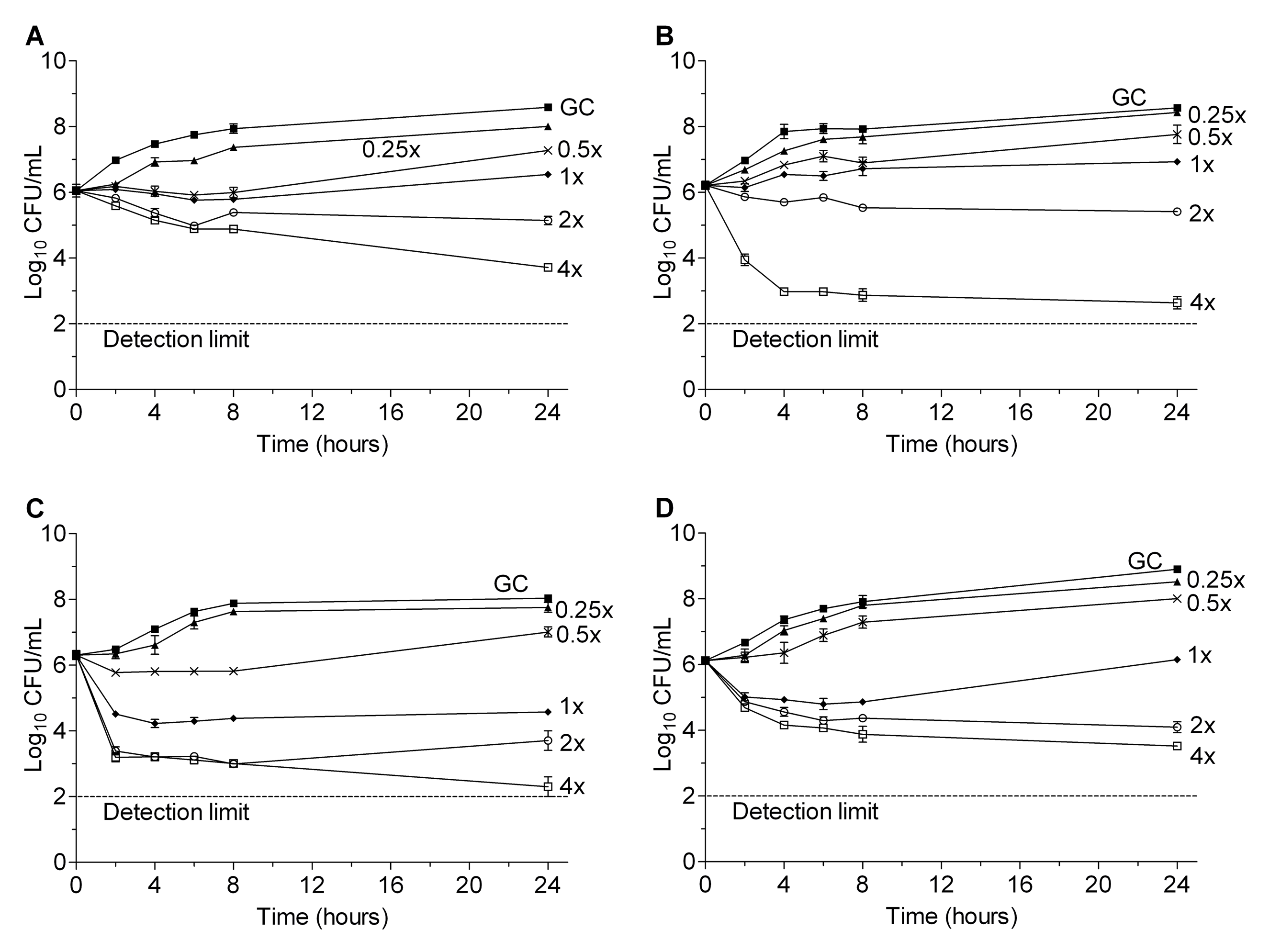

Supplement: Figure S2 — Activity of SNH at different concentrations in time-kill analysis against MSSA strains. A. MSSA 7–3 (MIC = 32 µg/mL); B. MSSA 7–4 (MIC = 32 µg/mL); C. MSSA 8–8 (MIC = 32 µg/mL); D. ATCC29213 (MIC = 32 µg/mL); ▪, GC, growth control; ▴, 1/4×MIC; ×, 1/2×MIC; ⧫, 1×MIC; ○, 2×MIC; □, 4×MIC. (TIF) [file pone.0068053.s002.tif]

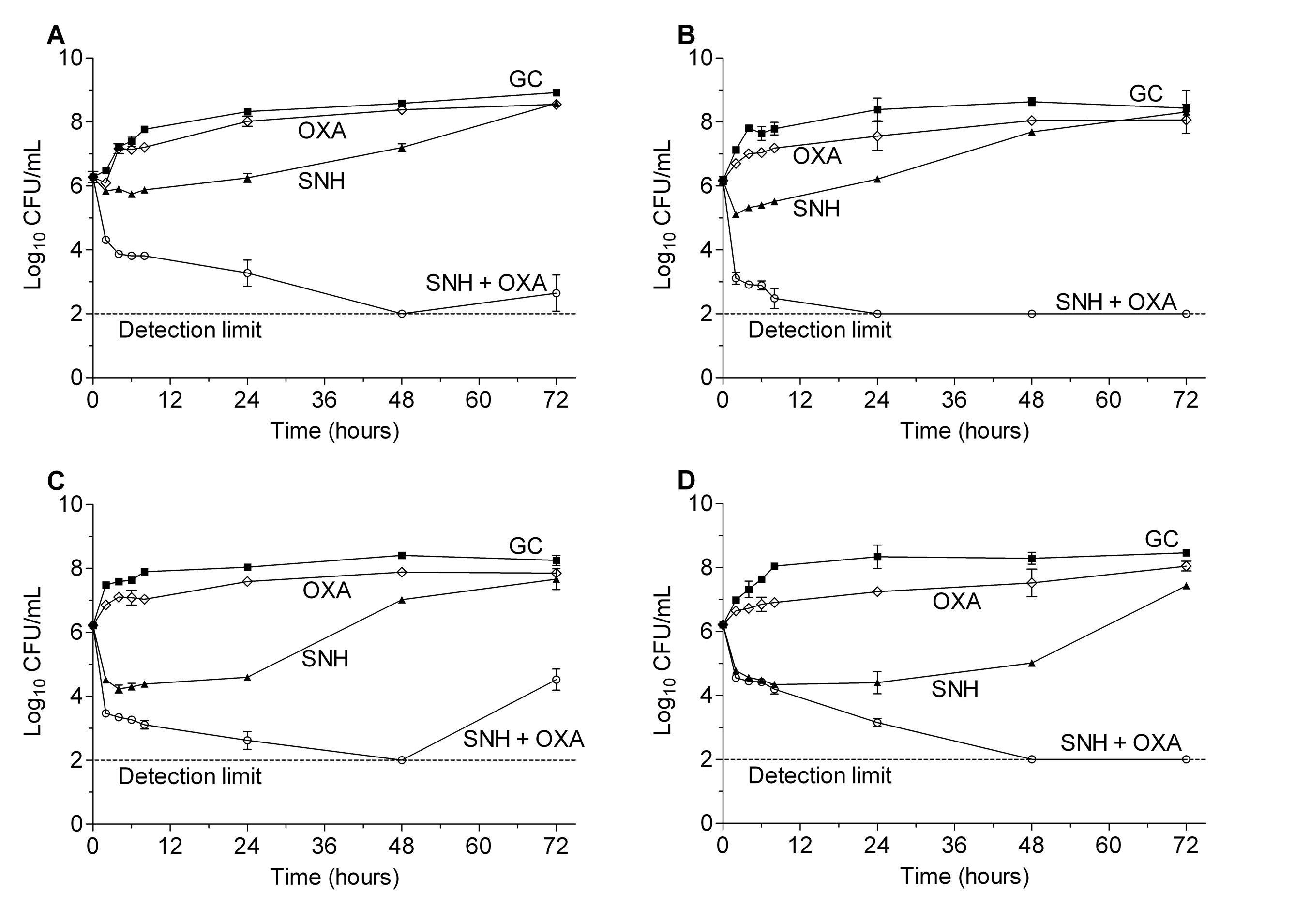

Supplement: Figure S3 — Sub-MIC levels of SNH and oxacillin alone and in combination against MSSA strains. A. MSSA 7-3, 1/2×MIC SNH-1/4×MIC OXA(MIC of SNH = 32 µg/mL, MIC of OXA = 0.5 µg/mL); B. MSSA 7-4, 1/2×MIC SNH-1/4×MIC OXA (MIC of SNH = 32 µg/mL, MIC of OXA = 0.5 µg/mL); C. MSSA 8-8, 1/2×MIC SNH-1/4×MIC OXA (MIC of SNH = 32 µg/mL, MIC of OXA = 0.5 µg/mL); D. ATCC29213 1/2×MIC SNH-1/4×MIC OXA (MIC of SNH = 32 µg/mL, MIC of OXA = 0.5 µg/mL); ▪, GC, growth control; ▴, SNH; ◊, OXA; ○, combination of SNH and OXA. (TIF) [file pone.0068053.s003.tif]
