# Supplementary material for: In Vitro Activity of Sodium New Houttuyfonate Alone and in Combination with Oxacillin or Netilmicin against Methicillin-Resistant Staphylococcus aureus
Source: PLoS One. 2013 Jul 2;8(7):e68053. doi: 10.1371/journal.pone.0068053 (PMC3699466; doi:10.1371/journal.pone.0068053)
Supplement: Table S1 — MICs and FICI of SNH and eight antibiotics against 12 MRSA strains in checkerboard assay. (PDF) [file pone.0068053.s004.pdf]

Table S1. MICs and FICI of SNH and eight antibiotics against 12 MRSA strains in checkerboard assay

Strain	origin	SCC *mec* type	MIC in single use (μg/mL)									FICI<br>MIC in combination (μg/mL)							
			SNH	OXA	CEF	MEM	VAN	LVX	MIN	NET	SXT	SNH/OXA	SNH/CEF	SNH/MEM	SNH/VAN	SNH/LVX	SNH/MIN	SNH/NET	SNH/SXT
MRSA 5-01	Pneumonia	III	32	512	256	32	1	32	8	16	0.5/9.5	0.28	0.31	0.25	1	1	0.63	0.38	0.75
												8/16	8/16	4/4	16/0.5	16/16	16/1	4/4	8/(0.25/4.8)
MRSA 5-16	Upper respiratory tract infection	III	16	512	256	64	1	4	8	32	1/19	0.38	0.38	0.31	0.63	1	0.75	0.25	0.5
												4/64	4/32	4/4	2/0.5	8/2	8/2	2/4	4/(0.25/4.8)
MRSA 5-20	Skin and soft tissue wounds	II	32	128	64	16	1	8	8	16	0.5/9.5	0.38	0.38	0.25	1	0.53	0.75	0.25	0.75
												8/16	8/8	4/2	16/0.5	1/4	16/2	4/2	8/(0.25/4.8)
MRSA 5-33	Pneumonia	III	32	512	256	32	1	16	8	16	0.5/9.5	0.56	0.31	0.38	1	2	0.63	0.38	0.63
												16/32	8/16	8/4	16/0.5	32/16	16/1	8/2	4/(0.25/4.8)
MRSA 6-03	Bacteremia	III	16	16	16	8	1	8	0.5	4	0.5/9.5	0.5	0.63	0.38	2	1	0.63	0.5	2
												4/4	8/2	4/1	16/1	8/4	2/0.25	4/1	16/(0.5/9.5)
MRSA 6-29	Bacteremia	III	32	256	128	32	1	32	0.5	8	1/19	0.31	0.38	0.25	1	1	0.63	0.5	0.75
												8/16	8/16	4/4	16/0.5	16/16	16/0.06	8/2	16/(0.25/4.8)
MRSA 8-36	Pneumonia	III	32	512	256	64	1	32	8	64	1/19	0.31	0.31	0.19	0.75	1	0.63	0.25	0.63
												8/32	8/16	4/4	16/0.25	16/16	16/1	4/8	4/(0.5/9.5)
MRSA 8-45	Urinary tract infection	III	32	512	256	32	1	8	8	16	0.5/9.5	0.5	0.63	0.25	0.75	0.75	0.63	0.25	1
												8/128	16/32	4/4	16/0.25	4/4	16/1	4/2	16/(0.25/4.8)
MRSA 8-48	Upper respiratory tract infection	III	32	512	256	128	1	32	8	1	1/19	0.5	0.5	0.16	1	2	0.75	0.5	0.38
												8/128	8/64	4/4	16/0.5	32/32	16/2	8/0.25	4/(0.25/4.8)
MRSA 8-52	Skin and soft tissue wounds	III	64	256	256	64	1	0.25	0.25	2	0.125/2.4	0.38	0.38	0.31	0.75	0.75	0.5	0.38	0.53
												16/32	16/32	16/4	16/0.5	16/0.125	16/0.06	8/0.5	2/(0.06/1.2)
ATCC33591	ATCC	III	32	256	32	16	1	0.25	8	4	1/19	0.31	0.5	0.25	1	0.75	0.63	0.25	0.63
												8/16	8/8	4/2	16/0.5	8/0.125	16/1	4/0.5	4/(0.5/9.5)
Mu 50	ATCC	II	64	512	128	32	8	16	8	16	0.25/4.8	0.5	0.56	0.38	0.75	1	0.5	0.5	1
												16/128	32/8	16/4	16/4	32/8	16/2	16/4	32/(0.13/2.4)
