# Supplementary material for: In Vitro Activity of Sodium New Houttuyfonate Alone and in Combination with Oxacillin or Netilmicin against Methicillin-Resistant Staphylococcus aureus
Source: PLoS One. 2013 Jul 2;8(7):e68053. doi: 10.1371/journal.pone.0068053 (PMC3699466; doi:10.1371/journal.pone.0068053)
Supplement: Table S2 — Susceptibility of 20 MSSA strains to SNH and oxacillin. (PDF) [file pone.0068053.s005.pdf]

Table S2. Susceptibility of 20 MSSA strains to SNH and oxacillin

Antimicrobial agent	MIC of reference strain ATCC 29213	MIC of 20 clinical isolates[a] (μg/mL)			Susceptible/total (% Susceptibility)
		Range	50%	90%	
SNH	32	16-32	32	32	NA[b]
OXA	0.5	0.25-0.5	0.5	0.5	20/20 (100%)

a. Origins of the 20 clinical isolates: Upper respiratory tract infection, 8 strains; pneumonia, 4 strains; skin and soft tissue wounds, 8 strains.

b. NA, not applicable
